# Sex-related Variability of White-Matter Tracts is Robust to Tractography Methodology

**DOI:** 10.21203/rs.3.rs-9439593/v1

**Published:** 2026-04-22

**Authors:** Matthew Amandola, Bastien Herlin, Michael E. Kim, Simon Vandekar, Ivy Uszynski, Bennett Landman, Cyril Poupon, Kurt G. Schilling

**Affiliations:** Vanderbilt University Institute of Imaging Science; Université Paris-Saclay, CNRS, CEA; Vanderbilt University; Vanderbilt University Medical Center; Université Paris-Saclay, CNRS, CEA; Vanderbilt University Institute of Imaging Science; Université Paris-Saclay, CNRS, CEA; Vanderbilt University Institute of Imaging Science

**Keywords:** tractography, reproducibility, sex-effects, microstructure

## Abstract

Diffusion tractography is the prominent *in-vivo* technique to study and investigate white-matter pathways in the human brain. While tractography is a powerful method, recent work suggests that different tractography methods can produce strikingly different representations of the same white-matter pathway. This multitude of differing options and diverging pipelines makes tractography related group-effects difficult to generalize, as it is currently unclear whether group-level inferences made using one tractography pipeline can be expected to hold when a different, equally defensible pipeline is applied to the same data. Here, we test the generalizability of sex-related changes on tractography-derived features by analyzing the exact same datasets with two equally reasonable pipelines which differ in model fitting, tractography reconstruction, and microstructure and volumetric analysis.

We found that despite differences in analysis, the resulting patterns and biological interpretations of sex effects rarely disagreed across methods. Microstructural effects between methods were remarkably consistent between protocols, only displaying one significant disagreement out of 343 comparisons (.29%). However, discrepancies were more common among volumetric effects, displaying 24% significant disagreement. Moreover, we found that reconstruction methods are differentially sensitive to tractography-derived features, as bundles derived from targeted tractography were much more sensitive to volumetric effects than tractogram-based tractography, potentially explaining the volumetric discrepancy between methods.

This study indicates that reasonable methodological choices are unlikely to lead two investigators to fundamentally opposing conclusions about sex differences in white-matter, and that the robustness of tractography findings is similar to established fields of science. More broadly, this study presents an optimistic outlook on the future of tractography, as it provides an empirical benchmark for reproducibility and bolsters confidence in the generalizability and robustness of tractography-derived findings.

## 1. Introduction

Diffusion tractography allows investigators to study previously inaccessible white-matter pathways *in-vivo*, and has become the premier method for categorizing the neuroanatomical characteristics of human white-matter. Bundle segmentation using tractography has contributed to things like identifying new pathways (Catani et al., 2013), cross-validating histological findings (Amandola et al., 2026; Girard et al., 2020; Thiebaut de Schotten et al., 2011), and further understanding white-matter’s role in cognition and disease (Forkel et al., 2022).

However, there is no consensus tractography processing pipeline, resulting in widely variable bundle segmentation protocols and techniques. As a result, recent studies have demonstrated that multiple different, yet equally reasonable, tractography methods can produce strikingly different representations of the same white-matter pathways (Maier-Hein et al., 2017; Schilling et al., 2021). This is due to the many different options at each step of the tractography workflow: reconstruction and model fitting (i.e., model choice and fitting strategy), tractography and bundle segmentation process (i.e., choice in fiber orientation reconstruction, streamline propagation algorithm and bundle segmentation technique), and quantitative analysis (i.e., mean extraction and density-weighted extraction). Moreover, results from these varying techniques are compounded by the prevalence of false positives trajectories in tractography (Maier-Hein et al., 2017). As a result, it is unclear whether group-level inferences made using one tractography pipeline can be expected to hold when a different, equally defensible pipeline is applied to the same data.

While this is a crucial realization, this proposes another important question: do these variabilities between pipelines lead to contradictory interpretations associated with these pathways? To answer this, we were motivated by recent work utilizing tractography to study an exemplar biological effect: sex-related differences (Herlin et al., 2024). Our goal here is to test if the same study employing a different tractography pipeline would lead to identical conclusions, highlighting the importance of understanding the potential effects of tractography methodology on the generalizability of study results.

In the current study, we ask whether two different automated bundle segmentation methods applied to the same exact dataset lead to consistent conclusions about white-matter pathways. In direct collaboration with (Herlin et al., 2024), we directly tested the reproducibility and generalizability of tractography-derived sex differences in white-matter micro- and macrostructural features using two widely used automated tractography techniques. Both laboratories independently processed the Human Connectome Project Young Adult (HCP) (Van Essen et al., 2012) dataset using their own bundle segmentation methods, extracted a common set of microstructural features, and tested for sex-related differences. We then quantified agreement between pipelines in terms of statistical significance and effect directionality, asking whether two automated tractography methods lead to convergent versus contradictory conclusions. We found that despite substantial differences in the definitions, shapes, and spatial extents of the reconstructed bundles, both the resulting patterns of sex effects and, critically, the biological interpretation of microstructural effects were remarkably congruent, whereas volumetric effects are more likely to diverge. This work provides an optimistic and nuanced outlook on the robustness and interpretability of tractography-derived findings.

## 2. Methods

### 2.1 Dataset

**Figure 1:**
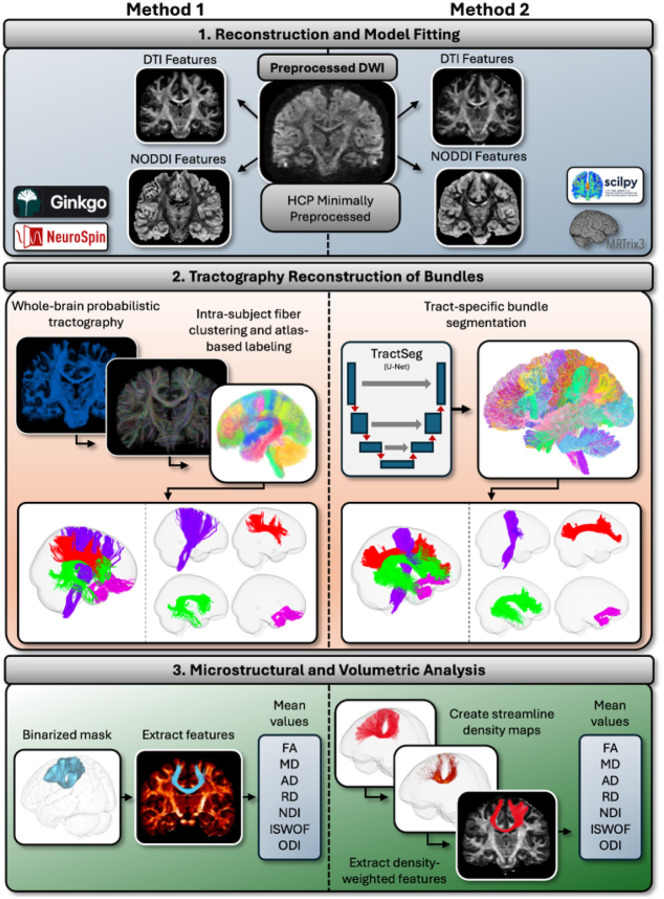
Pipeline overview between the whole-brain tractogram method (Method 1) vs the bundle-specific bundle segmentation method (Method 2).

Both labs used the exact same dataset: We analyzed 1,065 healthy young adults (575 women, 490 men; age range 22–35 years) from the Human Connectome Project Young Adult (HCP-YA) minimally preprocessed cohort (Glasser et al., 2013), which had performed intensity normalization of the mean b0 image, EPI distortion correction, EDDY correction, and gradient nonlinearity correction to the diffusion data. Differences in processing start to arise at: 1. dMRI processing, 2. Tractography reconstruction, and 3. Microstructural and volumetric analysis of the tracts (Fig. 1), detailed below.

#### 2.1.1 Reconstruction and Model Fitting

Method 1 is an in-house pipeline designed by the Neurospin team (https://framagit.org/cpoupon/gkg) based on the Ginkgo toolbox. This toolbox calculates the Diffusion Tensor Imaging (DTI) model (Basser et al., 1994) and the Neurite Orientation Dispersion and Density Imaging (NODDI) model (H. Zhang et al., 2012) on the data. This toolbox also calculates the Orientation Distribution Functions (ODF) for each voxel using the Q-Ball model (Descoteaux et al., 2007). Both NODDI and ODF were calculated using all three diffusion shells. DTI metrics were calculated using only the b = 1000 s/mm^2^ shell, using weighted least squares (WLS).

Method 2 employs MRTrix3 (Tournier et al., 2019) to fit the DTI model on the data using all volumes with b = 1000 s/mm^2^, using iterated weighted least-squares (IWLS). The NODDI model is then fit to the data using the scilpy toolkit (Renauld et al., 2026) using all three diffusion shells. Method 2 uses constrained spherical deconvolution (CSD) (Jeurissen et al., 2014) to calculate ODFs, contained within the tractography process (see 2.2.2 - Tractography Reconstruction).

Both methods calculate fractional anisotropy (FA), mean diffusivity (MD), axial diffusivity (AD), and radial diffusivity (RD) using their respective DTI-derived scalar map pipelines, as well as isotropic water fraction (ISOWF), neurite density index (NDI), and orientation dispersion index (ODI) using their respective NODDI pipelines.

#### 2.1.2 Tractography Reconstruction

For tractography reconstruction, Method 1 uses ODF maps to conduct whole-brain probabilistic tractography, resulting in a complete tractogram for each subject (Perrin et al., 2005). Then, a predefined deep white-matter atlas (Chauvel et al., 2023; Herlin et al., 2023) is coregistered to each subject’s native diffusion space from standard MNI (Montreal Neurological Institute) (Fonov et al., 2011) using the Advanced Normalization Tools (ANTs) toolbox (Avants et al., 2008) to first create the native-to-MNI transformation, and then the inverse MNI-to-native transformation, which is then applied to the deep white-matter atlas. Automated bundle segmentation from the white-matter atlas is then performed in the native diffusion space using the subject’s tractogram. Streamlines are then assigned to a possible 77 different tracts based on the white-matter atlas using pairwise distance. For full details on the mechanics of this automatic segmentation algorithm, see Herlin et al., 2024.

Method 2 uses Tractseg (Wasserthal et al., 2018), which automatically segments anatomically defined deep white-matter pathways. Tractseg uses MRTrix3 (Tournier et al., 2019) to compute the ODF peaks per voxel using CSD. Using a convolutional neural network model, and then performs bundle-specific tractography based on these ODF peaks, and outputs 72 predefined bundles of streamlines.

We identified 49 white-matter bundles these methods have in common. For a full list of bundles included in this analysis, see Table 1.

#### 2.1.3 Microstructural and Volumetric Analysis

Method 1 computes total brain volume (TBV) using Freesurfer (Fischl, 2012). Volume for each tract is then computed with a streamline density-weighted mask with a threshold of 5 fibers per voxel. Normalized volume (N. Vol.) is then calculated by dividing each tract’s volume by the corresponding individual’s TBV. Mean values for each tract and feature are then calculated after resampling the quantitative maps to 0.1mm via trilinear interpolation.

Method 2 also extracts streamline density-weighted averages of all path-feature pairs. For each tract, Method 1 uses the Scilpy toolkit (Renauld et al., 2026) to compute streamline density-weighted averages (note, in contrast to mean value within a binary mask) of DTI, NODDI, and volumetric features in native space. Similar to Method 1, tract volume was normalized by TBV, measured as estimated total intracranial volume and cerebral white-matter volume.

### 2.2 Statistical Analysis

#### 2.2.1 Sex Differences in Path-Feature Pairs

Both methods extract the following features for each subject and tract: AD, FA, MD, RD, NDI, ODI, ISOWF, volume, and normalized volume. Sex differences were tested independently for every tract-feature combination using Student’s t-tests comparing males and females, with Bonferroni correction applied to control for multiple comparisons across all tract-feature tests. With our 49 tracts and 9 features, there are a total of 4421comparisons in this study, which necessitates a significance threshold of *p* < .00001 following Bonferroni correction. However, in the original study (Herlin et al., 2024), significance was tested using a threshold of *p* < 0.000065 as there were more tract-feature pairs due to the current paper focusing on the tracts the two methods have in common. In order to compare group correspondence to the original analysis, we employed this more conservative threshold. We then computed effect size magnitudes for sex differences within each tract-feature pair using Cohen’s d test, with |d| < 0.2 meaning a negligible effect size, |d| = 0.2– 0.5 meaning small effect size, |d| = 0.5 – 0.8 meaning medium effect size, and |d| > 0.8 meaning large effect size.

#### 2.2.2 Agreement between Tractography Pipelines

We quantified agreement between the two methods on sex differences between tracts in features in their datasets. Agreement between methods was quantified at four levels, listed below:

- **Strict Agreement:** Both results statistically significant with the same direction, or neither results are significant
- **Soft Agreement:** Both results statistically significant with the same direction, or one significant with the other non-significant but same direction
- **Strict Disagreement:** Both results statistically significant with opposite directions
- **Soft Disagreement:** Both results statistically significant with opposite directions, or one significant with the other non-significant but opposite direction

Finally, to compare the sensitivity of the two methods, we employed a non parametric bootstrap with 10,000 iterations to estimate 95% confidence intervals (CI) for the difference in effect size magnitude between techniques for each feature, and considered sensitivity to differ significantly when the CI excluded zero.

## 3. Results

### 3.1 Methodology correspondence

Across most features and tracts, both methods agreed that males show higher values; FA was the sole exception, with both methods consistently indicating higher FA in females across tracts. To evaluate the generalizability of the sex effects of tractography-derived features, we calculated agreement and disagreement between the two methods. Across all 441 tract-feature comparisons, we observed strong correspondence between the two automated tractography techniques (Fig. 2). Between the two methodologies, 61.8% of tract-feature pairs reached strict agreement, as both pipelines yield either a significant sex effect in the same direction or a non-significant result. When the criterion was expanded to soft agreement, agreement increased to 86.7%, as the direction of the effect, irrespective of whether each pipeline’s result passed multiple comparison correction, was consistent between the two methodologies. Thus, in the vast majority of cases, both techniques pointed toward the same qualitative pattern of sex differences, even when only one pipeline reached formal significance.

Direct contradictions between techniques were rare. Strict disagreement occurred in only 2% of comparisons: out of 441 tract-feature pairs, there were 9 instances in which both methods produced statistically significant but directionally opposing effects. When expanded to soft disagreement to include any opposite directionality, regardless of significance, disagreement was 24.2%. These disagreements were typically characterized by very small effect sizes near zero in at least one pipeline, consistent with statistical noise rather than systematic reversals of effect. These results suggest that correspondence between tractography methodologies, regardless of reconstruction technique, algorithm, and feature extraction was exceptionally high.

Notably, the disagreement between methods was driven by volumetric features (Fig. 3). Specifically, while tract-volume sex-effects were fully in agreement, normalized volume effects frequently diverged. Microstructural features very rarely disagreed: out of 343 comparisons, only one tract-feature was significantly different between methods (.3%). However, when considering only volumetric effects, strict disagreement rose to 8.1%, with 8 tract-feature pairs significantly different between the two methods.

### 3.2 Methodological Sensitivity

**Figure 4:**
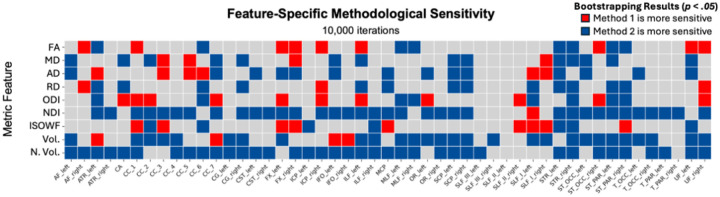
Feature-Specific Methodological Sensitivity. Bootstrap analysis for 10,000 iterations was conducted to calculate 95% confidence intervals in order to test if one method was significantly more sensitive for a given metric feature than another. Orange = method 1 (whole-brain tractogram) is more sensitive; blue = method 2 (Tractseg) is more sensitive.

We also examined whether one method was systematically more sensitive to sex differences in particular features (Fig. 4). In this case, sensitivity corresponds to a significantly different effect size between methods revealed by bootstrapping. For microstructural measures, the bootstrap analysis of effect size magnitude revealed that with the exception of NDI, there was no differentiable pattern in feature sensitivity between the two methods, with Method 1 significantly more sensitive in 42 features, and Method 2 more sensitive in 48 features. When considering NDI, Method 2 was significantly more sensitive, making the totals 43 for Method 1 and 83 for Method 2, as NDI alone contributed 35 additional tract-feature pairs for Method 2

For volumetric effects, Method 2 was considerably more sensitive, as it was significantly more sensitive in 65 tract-feature pairs, compared to 3 tract-feature pairs for Method 1. This difference in sensitivity may partly explain the considerable divergence in sex effects between the two methods, as normalized volume displayed the most divergence between all the features.

Taken together, these results indicate that while tractography pipelines can differ in how often they detect statistically significant sex effects for particular features, the qualitative conclusions drawn about the presence and direction of sex differences in white-matter microstructure are consistent across methods when applied to the same high quality dataset, though volumetric effects are still seem quite sensitive to methodology.

## 4. Discussion

Correspondence between tractography methods is critical for interpreting diffusion MRI studies that rely on automated bundle reconstructions. It is crucial that investigators understand whether observed group differences, such as sex effects, are robust properties of white-matter organization or artifacts of a particular processing pipeline. This issue is especially salient for tractography, which remains vulnerable to spurious streamlines and lacks an absolute anatomical ground truth (Dyrby et al., 2007). Indeed, tractography is a complex, multifaceted analysis, where segmented bundles are impacted by each step of the processing pipeline (Maier-Hein et al., 2017), highlighting the significance in understanding pipeline variability’s effect on potential biological interpretations.

In this large sample of HCP-YA participants, we found encouraging evidence that tractography-derived sex differences in white-matter microstructure are generalizable across two independent, state of the art automated pipelines. Importantly, biological interpretation remained consistent between methodologies, with strict agreement in both significance and directionality observed for roughly two thirds of tract-feature comparisons, a rate comparable to (Brodeur et al., 2026), and in some cases exceeding (Tyner et al., 2026), cross-pipeline reproducibility reported in other scientific fields. Additionally, overall agreement in the direction of effect was 90%, with only one strict microstructural disagreement out of 343 possible comparisons. Encouragingly, while the motivator of this study was work by Herlin and colleagues (2024), it is important to note that these results are in consensus and are further supported by previous sex-related tractography literature, which typically finds that women exhibit higher FA in these long-range bundles (Herlin et al., 2024; Kochunov et al., 2015; Raikes et al., 2025). Notably, this includes recent work using altogether different methodology by employing multitensor tractography and using unweighted mean metric values (F. Zhang et al., 2025), and found congruent sex-related findings in tracts such as the arcuate fasciculus, cerebellar peduncles, corticospinal tract, and the thalamic radiations.

While microstructural effects were remarkably congruent, our findings suggest that volumetric features were sensitive to methodology, comprising 8 of the 9 strict disagreements between methods, and suggesting that women may show a higher volume of white-matter compared to gray-matter, as this was true across bundles. This may reflect the inconsistent nature of sex-related effects of white-matter volume in past literature, as in the past, studies report that men display higher white-matter to gray-matter ratio (Kanaan et al., 2012). However, more recent papers correcting for TBV suggest that there is no differentiable trend in white-matter volume between men and women (Ramzanpour et al., 2023; Muer et al., 2024), suggesting our findings may stem from the given reconstruction technique. These differences may also be explained by methodological differences in sensitivity to volumetric features: Method 2 was significantly more sensitive in volume, normalized volume, and NDI, where virtually all disagreements between methods arose. Therefore, researchers should consider which technique they use when probing specific pathways or features, particularly if their primary goal is maximizing sensitivity for a given metric.

While the current study focuses on sex-effects, we believe our results are overall optimistic for tractography, and are comparable to many scientific fields as a whole: they indicate that reasonable methodological choices may not always lead to perfect replication of results, but that they are unlikely to lead two investigators to fundamentally opposing conclusions about sex differences in white-matter microstructure. More broadly, this work provides an empirical benchmark for cross-pipeline reproducibility and gives tractography researchers greater confidence in the generalizability and robustness of their findings.

## Figures and Tables

**Table 1 –:** List of tracts shared between pipelines

Abbreviation	Full Tract Name
CA	Anterior Commissure
CC_1	Corpus Callosum: Rostrum
CC_2	Corpus Callosum: Genu
CC_3	Corpus Callosum: Rostral Midbody
CC_4	Corpus Callosum: Anterior Midbody
CC_5	Corpus Callosum: Posterior Midbody
CC_6	Corpus Callosum: Isthmus
CC_7	Corpus Callosum: Splenium
MCP	Middle Cerebellar Peduncle
AF_left	Left Arcuate Fasciculus
AF_right	Right Arcuate Fasciculus
ATR_left	Left Anterior Thalamic Radiation
ATR_right	Right Anterior Thalamic Radiation
CG_left	Left Cingulum
CG_right	Right Cingulum
CST_left	Left Corticospinal Tract
CST_right	Right Corticospinal Tract
FX_left	Left Fornix
FX_right	Right Fornix
ICP_left	Left Inferior Cerebellar Peduncle
ICP_right	Right Inferior Cerebellar Peduncle
IFO_left	Left Inferior Fronto-Occipital Fasciculus
IFO_right	Right Inferior Fronto-Occipital Fasciculus
ILF_left	Left Inferior Longitudinal Fasciculus
ILF_right	Right Inferior Longitudinal Fasciculus
MLF_left	Left Middle Longitudinal Fasciculus
MLF_right	Right Middle Longitudinal Fasciculus
OR_left	Left Optic Radiation
OR_right	Right Optic Radiation
SCP_left	Left Superior Cerebellar Peduncle
SCP_right	Right Superior Cerebellar Peduncle
SLF_I_left	Left Superior Longitudinal Fasciculus I
SLF_I_right	Right Superior Longitudinal Fasciculus I
SLF_II_left	Left Superior Longitudinal Fasciculus II
SLF_II_right	Right Superior Longitudinal Fasciculus II
SLF_III_left	Left Superior Longitudinal Fasciculus III
SLF_III_right	Right Superior Longitudinal Fasciculus III
ST_OCC_left	Left Striato-Occipital Tract
ST_OCC_right	Right Striato-Occipital Tract
ST_PAR_left	Left Striato-Parietal Tract
ST_PAR_right	Right Striato-Parietal Tract
STR_left	Left Superior Thalamic Radiation
STR_right	Right Superior Thalamic Radiation
Striato_Central_Left	Left Striato-Central Tract
Striato_Central_Right	Right Striato-Central Tract
Striato_Frontal_Left	Left Striato-Frontal Tract
Striato_Frontal_Right	Right Striato-Frontal Tract
T_OCC_left	Left Thalamo-Occipital Tract
T_OCC_right	Right Thalamo-Occipital Tract
T_PAR_left	Left Thalamo-Parietal Tract
T_PAR_right	Right Thalamo-Parietal Tract
UF_left	Left Uncinate Fasciculus
UF_right	Right Uncinate Fasciculus
